# Supplementary material for: Third Molar Agenesis Is Associated with Facial Size
Source: Biology (Basel). 2021 Jul 12;10(7):650. doi: 10.3390/biology10070650 (PMC8301315; doi:10.3390/biology10070650)
Supplement: Supplementary file 1 [file biology-10-00650-s001.zip › biology-1279758-supplementary.pdf]

biology

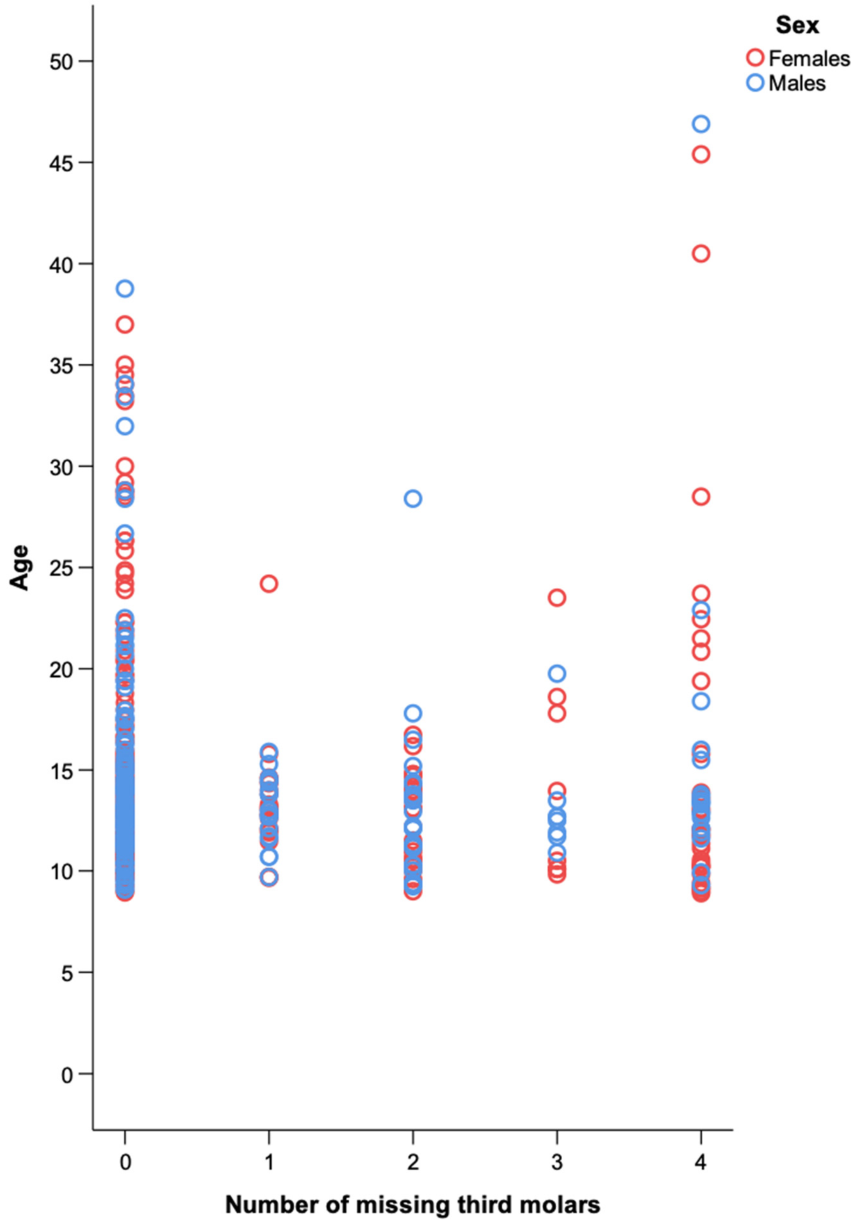

Figure S1. Sample distribution according to age, sex and number of missing third molars.

biology MDPI

a.) Entire cranial configuration

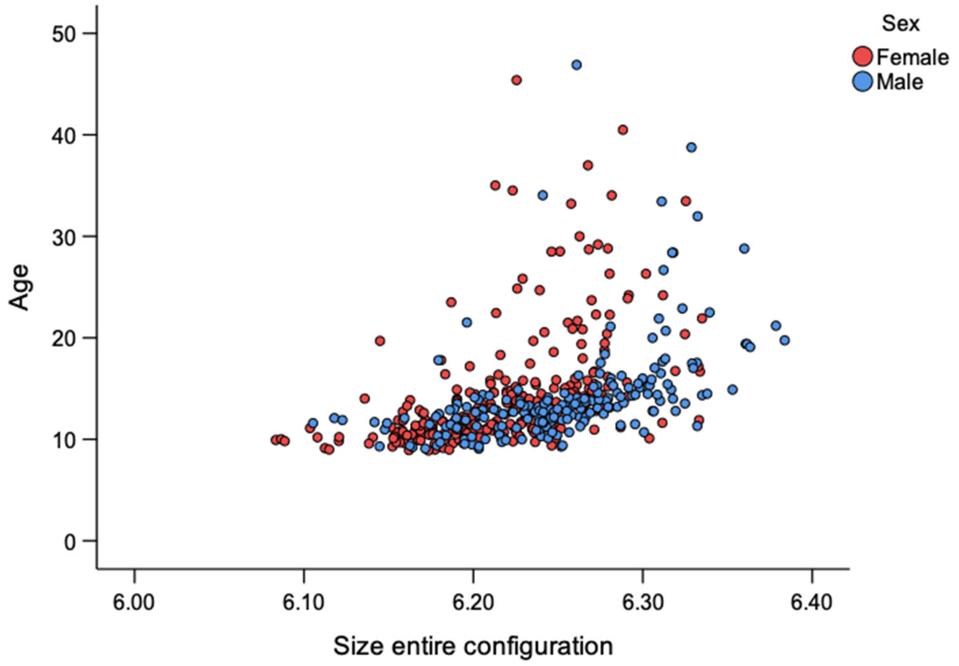

b.) Cranial base

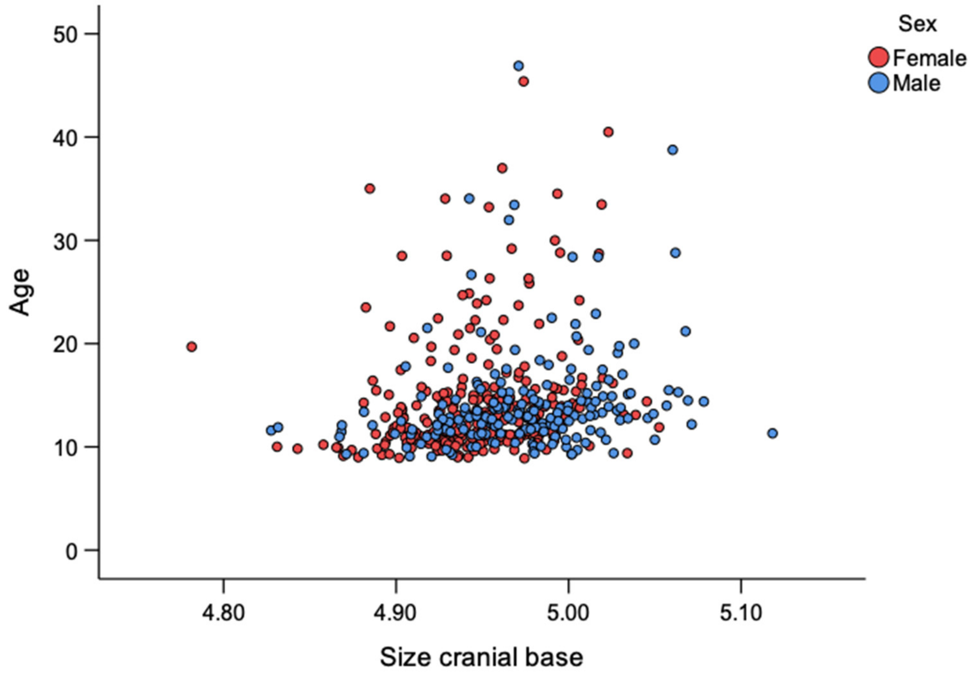

c.) Mandible

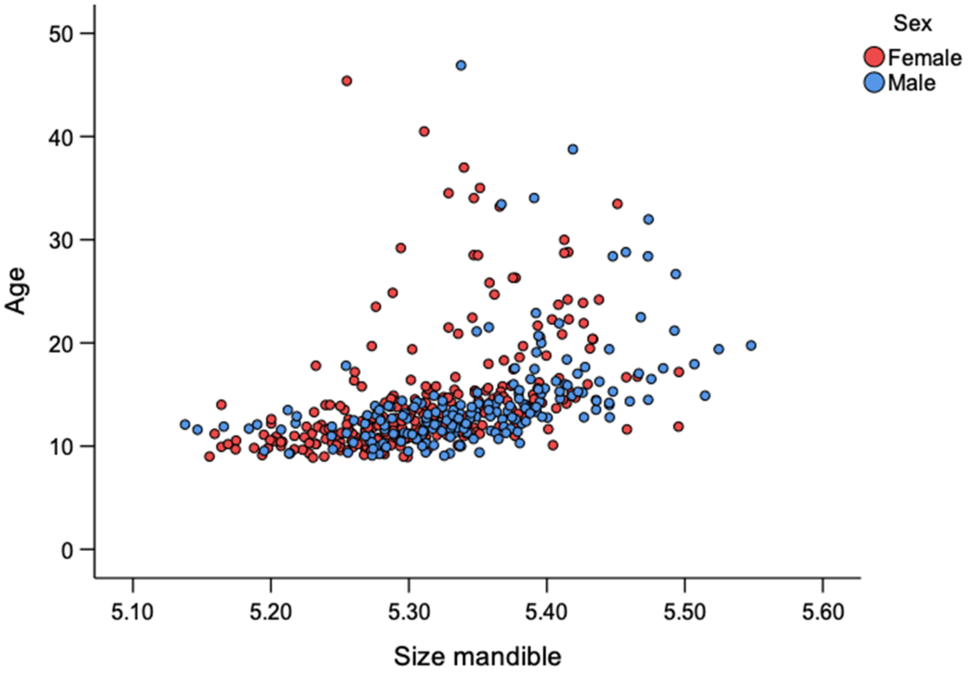

d.) Maxilla

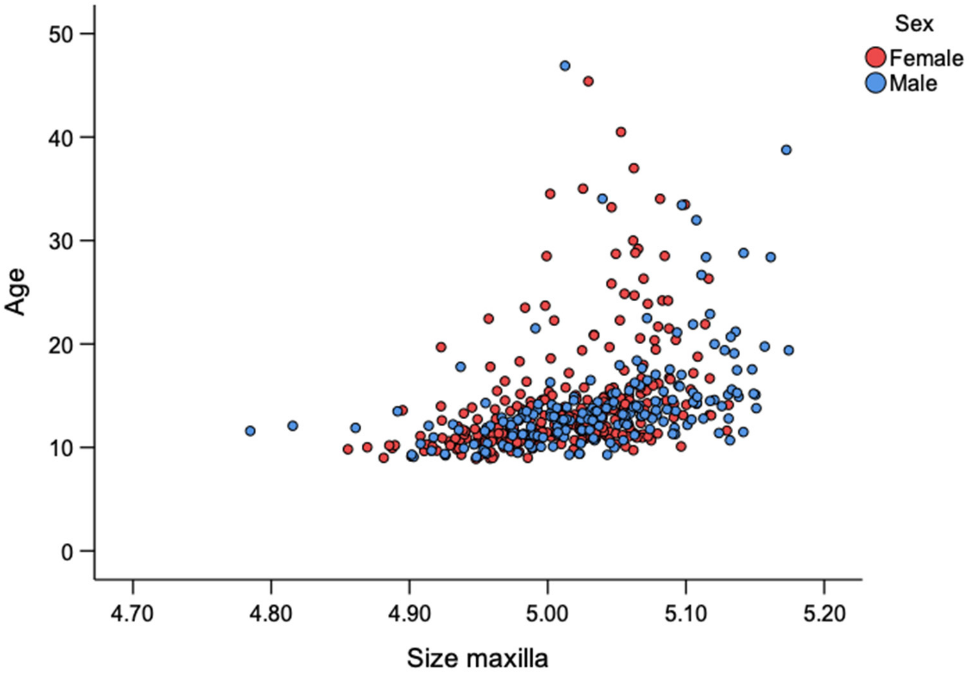

Figure S2: Scatterplots displaying size variability according to age.

a.) Entire cranial configuration

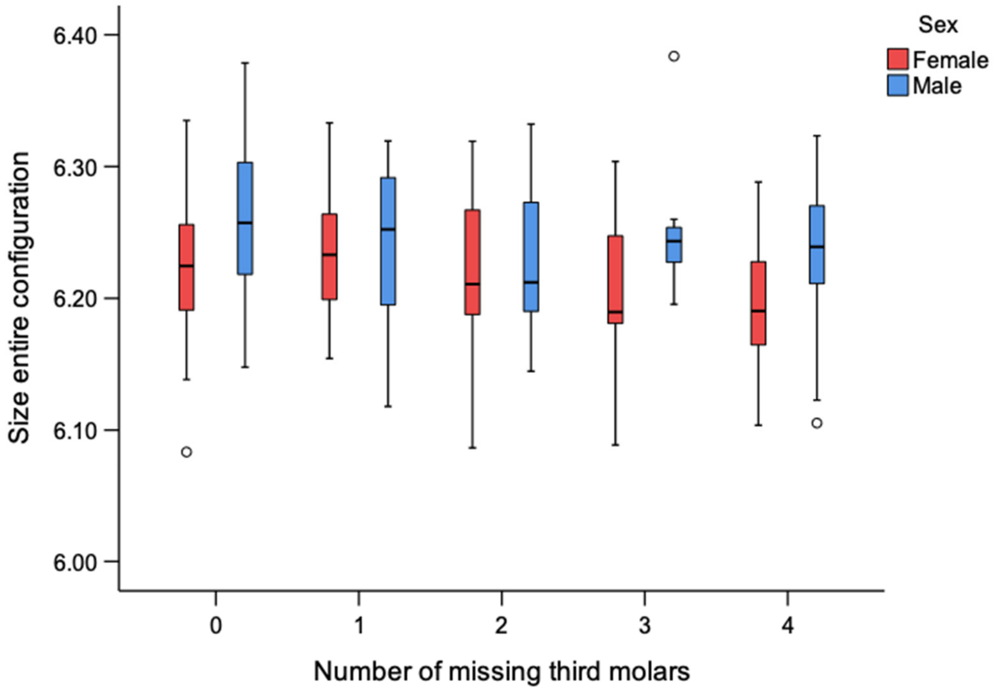

b.) Cranial Base

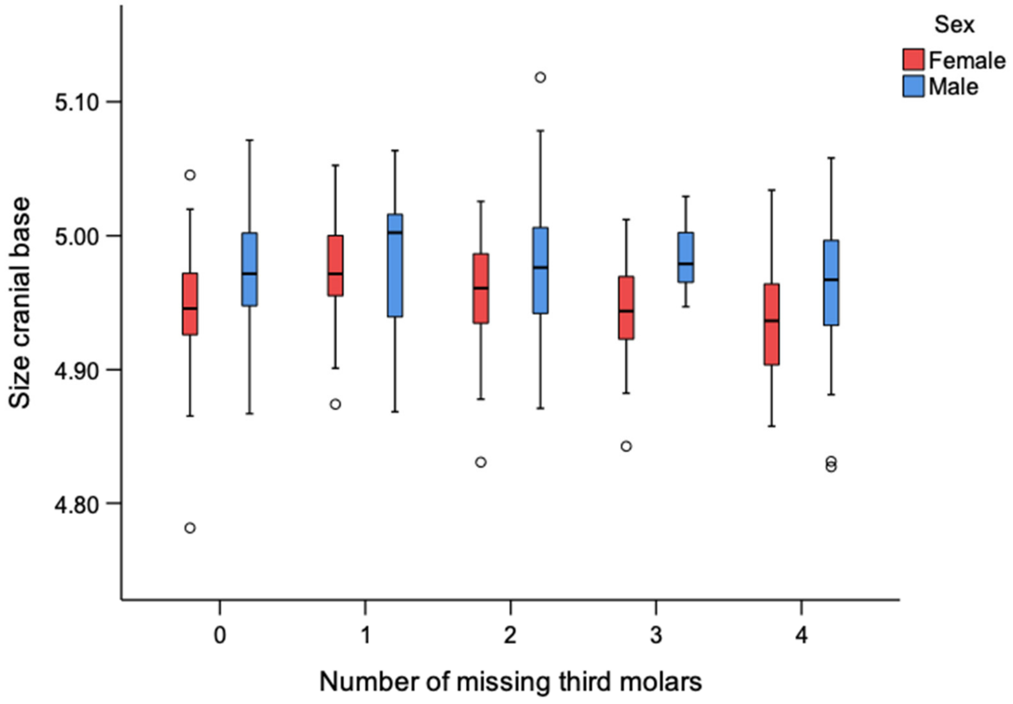

c.) Mandible

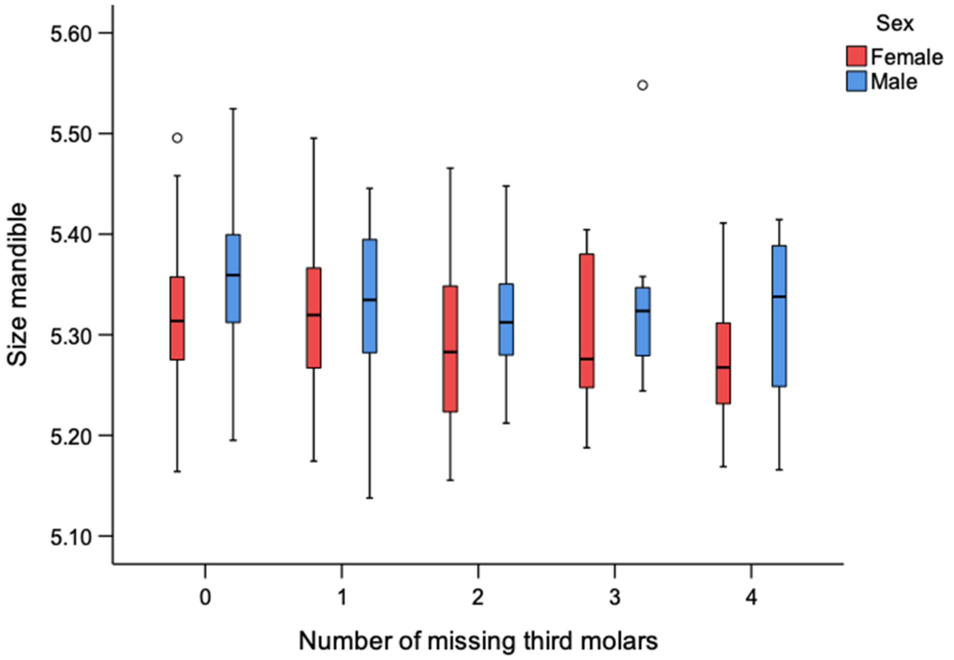

d.) Maxilla

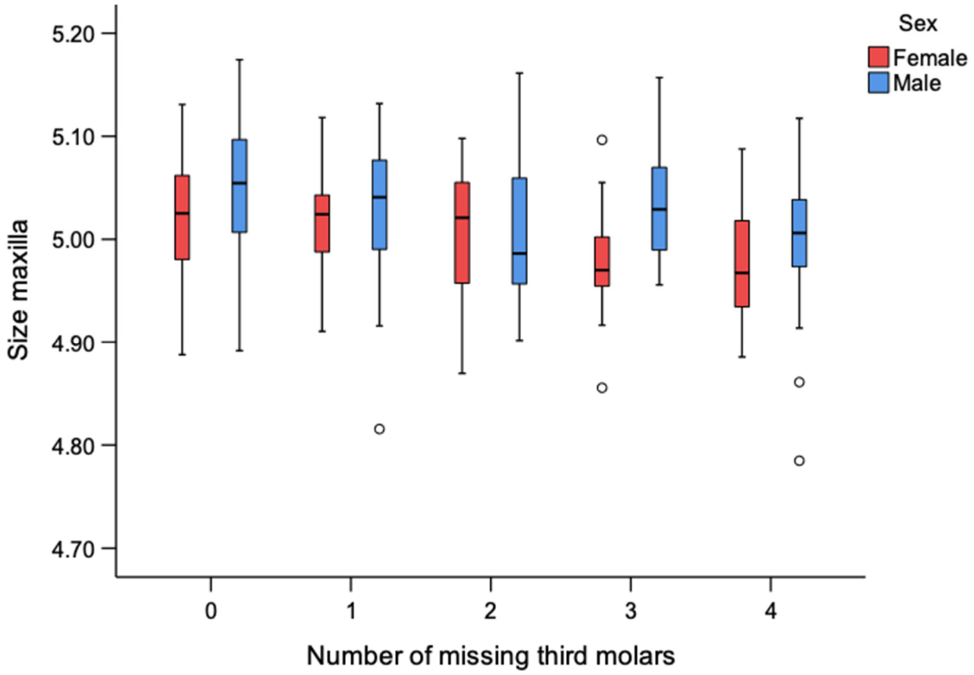

Figure S3: Box Plots showing size variability organized by number of missing third molars.
